# Differential Impairment of Interferon-*γ* Responses in Two Cases of Pulmonary Nontuberculous Mycobacterial Disease

**DOI:** 10.1155/2016/9165641

**Published:** 2016-11-16

**Authors:** William Rae, Yifang Gao, Efrem Eren, Rainer Döffinger, Ben Marshall, Anthony P. Williams

**Affiliations:** ^1^University Hospital Southampton NHS Foundation Trust, Southampton, UK; ^2^Academic Unit of Cancer Sciences, Faculty of Medicine, University of Southampton and Southampton NIHR Experimental Cancer Medicine Centre, Southampton, UK; ^3^Department of Clinical Biochemistry and Immunology, Addenbrooke's Hospital, Cambridge, UK; ^4^National Institute for Health Research, Cambridge Biomedical Research Centre, Cambridge, UK

## Abstract

Nontuberculous mycobacteria (NTMs) are weakly virulent intracellular pathogens that are common in food and water supplies. The persistent culture of these organisms in the setting of clinical infection warrants investigation of immune function. In cases of isolated pulmonary NTM (PNTM) disease, underlying immune defects have not been clearly identified. We present two patients with isolated PNTM infection who demonstrated differentially impaired IFN-*γ* production across a range of stimuli. These cases show that cellular IFN-*γ* responses may be defective in a proportion of patient suffering PNTM disease and that when assessing responses, the stimulant used in the testing is important to delineate defective cell populations. Impaired IFN-*γ* responses to IL-12 + BCG seem to be a poor prognostic indicator in PNTM disease and in these cases were not improved by adjuvant IFN-*γ*.

## 1. Introduction

Nontuberculous mycobacteria (NTMs) are weakly virulent intracellular pathogens that are common in food and water supplies. The persistent culture of these organisms in the setting of clinical infection warrants investigation of immune function. Pathogenic infections with NTMs are associated with defects in phagocytes, antigen presenting cells, natural killer (NK), and T-cells function [1]. “Mendelian susceptibilities to mycobacterial disease” (MSMD) comprises a rare group of monogenic primary immunodeficiencies that predispose an individual to disseminated NTM infection. MSMD is characterised by defects in the interleukin- (IL-) 12/interferon- (IFN-) *γ* pathway [2]. Immunosuppressive medication, HIV infection, metabolic diseases, and cytokine neutralising antibodies have all been identified as secondary immunodeficient states implicated in disseminated NTM infection [3]. However, in cases of isolated pulmonary NTM (PNTM) disease, underlying immune defects have not been clearly identified [4]. We present two patients with isolated PNTM infection who demonstrated differentially impaired IFN-*γ* production across a range of stimuli.

## 2. Case Presentations

### 2.1. Case  1

A 41-yr-old Caucasian male presented following a two-month history of productive cough, fevers, malaise, and weight loss. The patient had no prior history of recurrent infections or symptoms to suggest primary immunodeficiency. He had not received Bacille Calmette-Guérin (BCG) vaccination. HIV 1 and 2 serology and P24 antigen were negative.

A chest radiograph revealed right upper lobe consolidation and sputum cultured* Mycobacterium avium intracellulare* (MAI). Rifampicin, isoniazid, ethambutol, clarithromycin, moxifloxacin, and pyridoxine were commenced and following 18 months of treatment there had been no significant improvement in symptoms, and MAI continued to be cultured from sputum.

**Figure 1:**
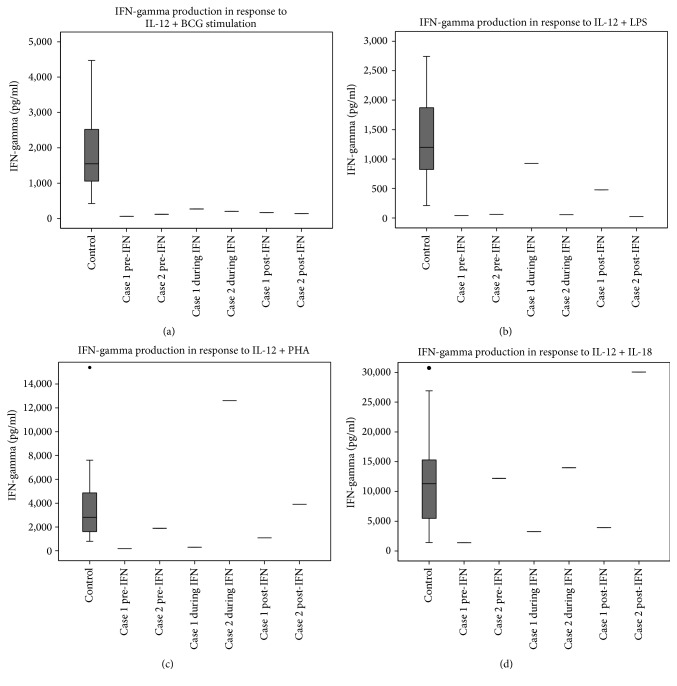
Box plots of whole blood IFN-*γ* production to stimulation with IL-12 ± BCG, ± phytohaemagglutinin, and ± IL-18, ± lipopolysaccharide. Whole blood was incubated with stimuli for 16 hours before supernatant was analysed for IFN-*γ*. Cases  1 and  2 were sampled at different time points: before commencing IFN-*γ* (pre-IFN), 3 months into IFN- *γ* therapy (during IFN), and 6 months after stopping IFN-*γ* therapy (post-IFN). Control box plot showing mean, interquartile range, and 1 standard deviation (whiskers) based on analysis of 27 healthy controls aged 20–60 yrs. Cases did not have detectable IFN-*γ* in the absence of stimuli (Supplementary Figure  1).

Immunological work-up showed a normal total lymphocyte count with a mild reduction of CD3+CD8+ T-cells, a slight reduction in IgM and protective Tetanus, total* Pneumococcal*, and* Haemophilus influenza* B IgGs (Supplementary Table  1 in Supplementary Material available online at http://dx.doi.org/10.1155/2016/9165641). Baseline (pre-IFN-*γ*)* ex vivo* whole blood stimulation showed reduced IFN-*γ* production compared with controls across all stimuli tested (Figure 1 and Supplementary Figure  1).

IFN-*γ* 100 mcg subcutaneously three times weekly was added alongside concurrent antimycobacterial therapy. During this period, the patient reported a reduction in sputum production and weight gain, but MAI continued to be cultured from sputum samples. After 3 months of IFN-*γ* treatment, there was an improvement in whole blood IFN-*γ* production to IL-12 + IL-18 and IL-12 + lipopolysaccharide stimulation, but persistently poor responses to IL-12 + BCG and IL-12 + phytohaemagglutinin (Figure 1 and Supplementary Figure  1). Screening for anti-cytokine autoantibodies against IFN-*γ*, IL-12, IL-6, and IL-17 was negative.

After 18 months of continued IFN-*γ* treatment, the patient developed side effects of myalgia and arthalgia, which resulted in the decision to stop IFN-*γ*. After 6 months off IFN-*γ* treatment, repeated assessment of IFN-*γ* production showed a sustained improvement in IFN-*γ* production to IL-12 + IL-18 and IL-12 + lipopolysaccharide (Figure 1). Despite these partial improvements in IFN-*γ* profile, the patient's condition continued to deteriorate with progressive pulmonary MAI infection, which ultimately resulted in death.

### 2.2. Case  2

A 53-year-old Caucasian male presented with persistent productive cough fevers and malaise. A chest radiograph demonstrated right upper lobe consolidation with background emphysematous changes. The patient had a past history of COPD and had been immunised with BCG without incident during adolescence. HIV 1 and 2 serology and P24 antigen were negative.

Bronchial washing identified* Mycobacterium Xenopi* and a computer tomography scan showed evidence of cavitation within the right upper lobe also consistent with active NTM infection. After 12 months of treatment with rifampicin, ethambutol, and ciprofloxacin, he remained unwell with dyspnoea, fatigue, and continuing* M. Xenopi* culture from sputum.

Immunological investigation revealed normal absolute lymphocyte counts, but an inverted CD4 : CD8 ratio of 0.7. An observed increase in IgA to 4.6 g/l was oligoclonal on immunoelectric-focusing. IgM was reduced at 0.3 g/l, but Tetanus,* Pneumococcal*, and* Haemophilus influenza* B IgG were protective (Supplementary Table  1). Whole blood assessment of IFN-*γ* production showed impaired responses to IL-12 + BCG and IL-12 + lipopolysaccharide, but IFN-*γ* responses to IL-12 + phytohaemagglutinin and IL-12 + IL-18 were comparable with controls (Figure 1). No anti-cytokine autoantibodies were detected in the patient's serum.

Adjuvant IFN-*γ* 100 mcg subcutaneous injection 3 times weekly was started in addition to the antimycobacterial agents. After 3 months of treatment, there had been no improvement in IFN-*γ* response to IL-12 + BCG or IL-12 + lipopolysaccharide (Figure 1). Over the next 12 months of continued IFN-*γ* therapy there was no isolation of* M. Xenopi* from sputum, yet he developed side effects of IFN-*γ* therapy involving muscle aches and flu-like symptoms resulting in the patient's decision to discontinue IFN-*γ*. Six months after stopping IFN-*γ*, but continuing antimycobacterial treatment, the pattern of low IFN-*γ* production to IL-12 + BCG and IL-12 + lipopolysaccharide remained unchanged from baseline (Figure 1). The patient's respiratory function gradually deteriorated, but he declined further IFN-*γ* therapy. Progressive chest sepsis and resultant lung parenchymal damage resulted in death.

## 3. Discussion

To date no reproducible immunological abnormalities have been identified in patients with isolated PNTM disease [5]. Impaired innate immunity due to lung parenchyma damage is believed to be the major predisposing factor for PNTM infection. PNTM disease is a serious cause of morbidity and mortality and has a rising incidence across populations [6, 7]. In cases with no past medical or family history suggestive of immunodeficiency, a deficiency in the production of IFN-*γ* has previously been noted prompting the adjuvant use of IFN-*γ* therapy [8–10]. Both patients demonstrated subtle defects in immunity, with a low IgM being common to both (Supplementary Table  1). The importance of this and other observations such as a reduction in CD3+CD8+ cells and inverted CD4 : CD8 ratio (Cases  1 and  2, resp.) is difficult to interpret in the setting of active infection. We report that the observation of impaired IFN-*γ* is dependent on the specific stimulant that is used in cases of PNTM infection and that impaired IFN-*γ* responses seem to be a poor prognostic indicator in PNTM infection. Adjuvant IFN-*γ* therapy appears to have limited benefit in isolated PNTM disease as it did not improve deficit responses. This pattern of differentially effected IFN-*γ* production during PNTM infection suggests that particular cell populations or pathways are impaired as opposed to a more generalised impairment.

## 4. Conclusion

These cases show that cellular IFN-*γ* responses may be defective in a proportion of patients suffering from PNTM disease and that the stimulant used in the testing is important to delineate the defective cell populations. Poor IFN-*γ* responses to IL-12 + BCG seem to be a poor prognostic indicator in PNTM disease and are unaffected by adjuvant IFN-*γ*.

## Supplementary Material

Supplementary Material contains basic immunological results for the patients at various timepoints, IFN-γ measurement in medium only (negative control), and TNFα production in response to LPS (positive cytokine control).
