# Supplementary material for: Differential Impairment of Interferon-γ Responses in Two Cases of Pulmonary Nontuberculous Mycobacterial Disease
Source: Case Reports Immunol. 2016 Nov 16;2016:9165641. doi: 10.1155/2016/9165641 (PMC5128696; doi:10.1155/2016/9165641)

**Supplementary Data**

**Supplementary data Table 1.**

Results of lymphocyte subsets, immunoglobulin, HIV, total Pneumococcal and Haemophilus IgG for Case 1 and 2. Normal ranges in brackets. (N/A = not available)

|  | **Case 1 pre IFNγ** | **Case 1 during IFNγ** | **Case 1 post IFNγ** | **Case 2 pre IFNγ** | **Case 2 during IFNγ** | **Case 2 post IFNγ** |
| --- | --- | --- | --- | --- | --- | --- |
| **White cell count x10^9^/l**  **(4.0-11.0)** | 8.0 | 7.5 | 12.6 | 10.3 | 12.3 | 9.1 |
| **Monocytes x10^9^/l**  **(0.2-1.0)** | 1.1 | 0.9 | 1.1 | 0.9 | 1.3 | 1.1 |
| **Lymphocytes cells/mm^3^**  **(1500 -4000)** | 1500 | 1800 | 1.1 | 1.7 | 2.1 | 1.8 |
| **CD3+ cells/mm^3^**  **(700-2100)** | 650 | 730 | N/A | 1330 | N/A | 1460 |
| **CD3+CD4+cells/mm^3^**  **(200-900)** | 510 | 570 | N/A | 710 | N/A | 580 |
| **CD3+CD8+ cells/mm^3^**  **(200-900)** | 140↓ | 140↓ | N/A | 540 | N/A | 840 |
| **CD19+ cells/mm^3^**  **(100-500)** | 450 | 570 | N/A | 150 | N/A | 90↓ |
| **NK cells (CD16/56+) cells/mm^3^**  **(60-600)** | 360 | 450 | N/A | 170 | N/A | 160 |
| **IgG g/l**  **(6-16)** | 7.0 | N/A | N/A | 12.2 | N/A | N/A |
| **IgA g/l**  **(0.8-2.8)** | 1.5 | N/A | N/A | 4.6↑ | N/A | N/A |
| **IgM g/l**  **(0.5-1.9)** | 0.3↓ | N/A | N/A | 0.3↓ | N/A | N/A |
| **Pneumococcal IgG u/ml**  **(>20)** | >340 | N/A | N/A | 56.7 | N/A | N/A |
| **Haemophilus B IgG u/ml**  **(>0.15)** | 0.58 | N/A | N/A | 0.21 | N/A | N/A |
| **Tetanus IgG iu/ml**  **(>0.01)** | >4 | N/A | N/A | >4 | N/A | N/A |
| **HIV 1 & 2 antibody/P24 Ag** | Negative |  |  | Negative |  |  |

**Supplementary Figure 1.**

IFN-γ detected in whole blood supernatant after incubation with cell medium only for 16 hours. Control values based on 27 healthy controls, bar indicates mean.


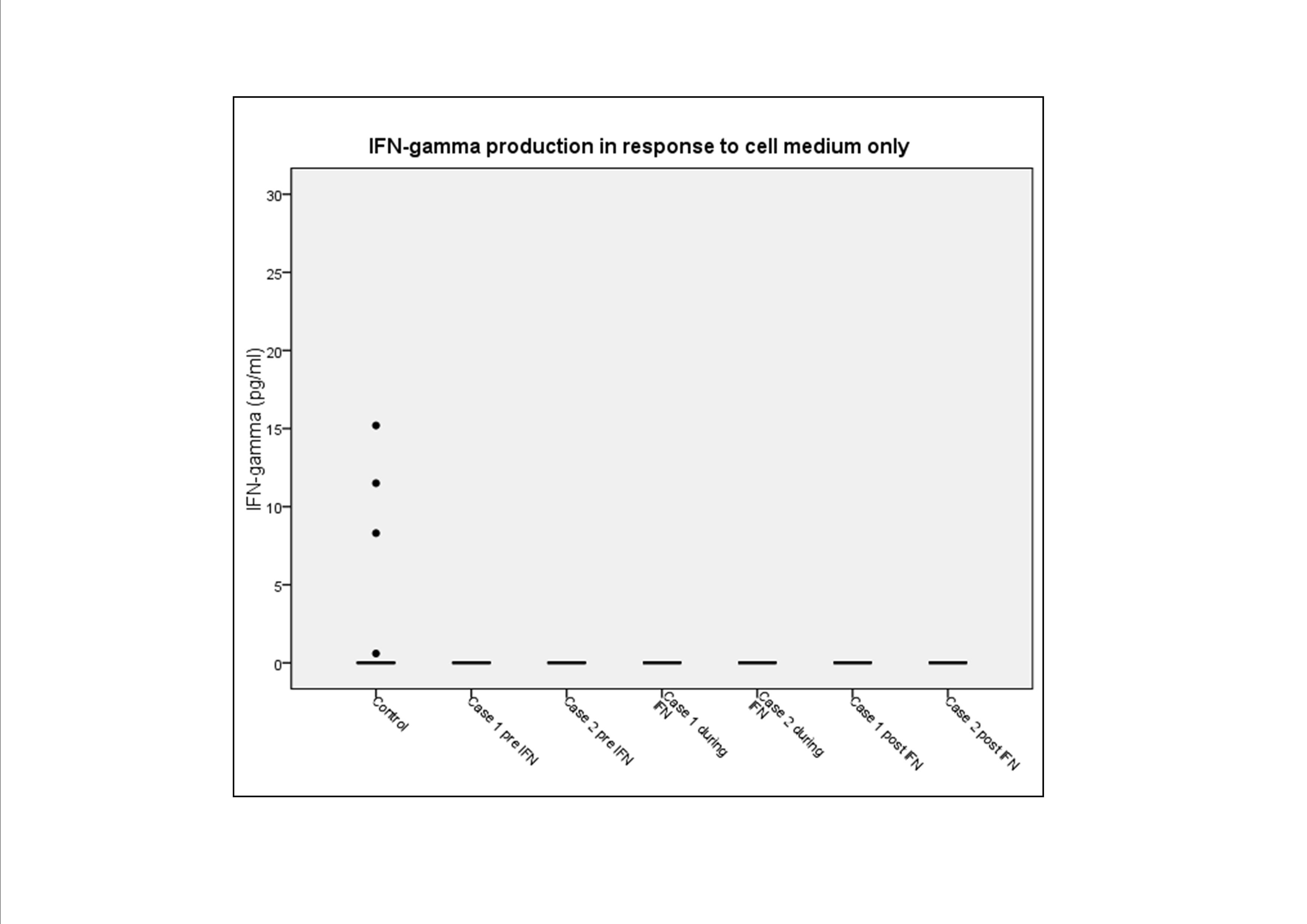


**Supplementary Figure 2.**

Patient lithium heparin whole blood samples are able to produce TNFα in response to LPS stimulation that is comparable with controls. Data suggests that the acquired deficit is specific to IFN-γ.


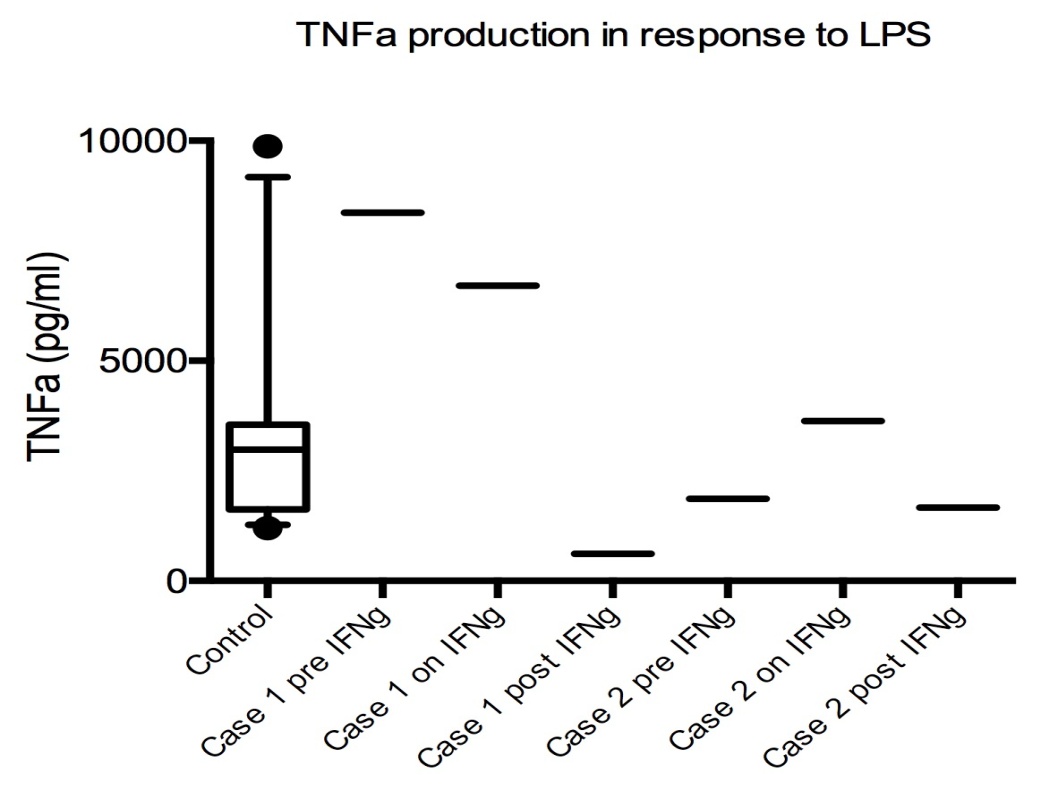

Supplement: Supplementary file 1 — Supplementary Material contains basic immunological results for the patients at various timepoints, IFN-γ measurement in medium only (negative control), and TNFα production in response to LPS (positive cytokine control). [file 9165641.f1.docx]
